# Association between plasma magnesium levels and glycolipid metabolism in a southern Chinese population: a cross-sectional study in Shenzhen

**DOI:** 10.3389/fendo.2026.1671459

**Published:** 2026-02-24

**Authors:** Meilin Li, Die Hu, Ziyang Zou, Jiaxin Chen, Dongju Zou, Yanwei Zhang, Jing-jun Han, Jinling Liu, Yanan Kuang, Baosen Yan, Jinquan Cheng, Ziquan Lv, Xiao Chen, Suli Huang

**Affiliations:** 1 School of Public Health, Shanxi Medical University, Taiyuan, China; 2 Immunization Section, Hefei Center for Disease Control and Prevention, Hefei, China; 3 Shenzhen Center for Disease Control and Prevention, Shenzhen, China; 4 School of Public Health, Shenzhen University Medical School, Shenzhen University, Shenzhen, China; 5 Department of Thoracic Surgery, The Eighth Affiliated Hospital, Sun Yet-Sen University, Shenzhen, China

**Keywords:** diabetes, glycolipid indicators, glycolipid metabolism, hyperlipidemia, magnesium

## Abstract

**Background:**

Numerous studies have delved into the relationship between magnesium (Mg) and metabolic diseases, however, the impact of Mg on novel glycolipid metabolic indicators remains largely unexplored. This study aims to conduct a comprehensive evaluation of the relationship between plasma Mg levels and glycolipid metabolism among a general population in Shenzhen, China.

**Methods:**

A cross-sectional study was performed in 1,429 adults who underwent medical check-ups at a hospital in Shenzhen, China. Plasma Mg levels were measured using inductively coupled plasma mass spectrometry (ICP-MS). To investigate the association between plasma Mg levels and glycolipid metabolism indicators, the multivariate linear and logistic regression models, along with the restricted cubic spline (RCS) model were employed.

**Results:**

Regarding to the glucose indicators, plasma Mg showed a negative linear association with the triglyceride-glucose index adjusted for Body Mass Index (TyG-BMI) and a positive linear association with the single point insulin sensitivity estimator (SPISE), while demonstrating a negative non-linear association with fasting blood glucose (FBG) and the metabolic score for insulin resistance (METS-IR). For lipid indicators, Mg exhibited a negative linear association with the non-high-density lipoprotein cholesterol to high-density lipoprotein cholesterol ratio (NHHR), low-density lipoprotein cholesterol (LDL-c), and high-density lipoprotein cholesterol ratio (HDL-c), while the LDL-c/HDL-c ratio showed a V-shaped non-linear relationship with Mg. Furthermore, the level of Mg exhibited a negative linear association with diabetes and a V-shaped non-linear association with hyperlipidemia.

**Conclusions:**

Mg plays a critical role in the glucose and lipid metabolism, particularly highlighting its association with the novel indicators for glycolipid metabolism. The results of our study need to be confirmed in large-scale prospective research in the future.

## 1 Introduction

Magnesium (Mg) is an indispensable mineral for maintaining human physiological homeostasis (1). Due to the frequent neglect of Mg’s critical role in human health in daily life, it is often referred to as the “forgotten” essential element. Mg plays a crucial role in reducing inflammation, facilitating glucose and insulin metabolism, enhancing endothelium-dependent vasodilation, and regulating blood lipid levels (2). The normal reference range for serum Mg is 0.75-0.95 mmol/L from the NHANES study (3). In the Chinese population, the normal range of serum Mg is 0.75-1.25 mmol/L (4). In healthy individuals, plasma Mg concentration is maintained at relatively stable levels, while it can be easily affected by disease states. Diabetic patients are particularly prone to Mg deficiency than no-diabetic people due to the increased urinary Mg excretion (5). Similarly, low Mg levels increase the risk of poor blood glucose control, thereby increasing the risk of cardiovascular disease (6).

Diabetes is widely prevalent in the Chinese population (7). Moreover, extensive research has been conducted on the link between Mg and diabetes (8). Several novel glycolipid metabolic indicators have been developed based on physiological and biochemical markers to assess the glycolipid metabolic status of individuals (9, 10). Epidemiological evidence has indicated that these novel metabolic indicators might offer more holistic assessment of the risk for metabolic diseases. For instance, the triglyceride glucose index (TyG) incorporates measurements of both fasting blood glucose (FBG) and triglycerides (TG) levels together. Extreme low or high level of TyG led to an increased risk of diabetes and cardiovascular death (11). The metabolic score for insulin resistance (METS-IR) is significantly correlated with the content of ectopic fat accumulation in the viscera and liver, which is an early indicator of insulin resistance (12). The non-high-density lipoprotein cholesterol to high-density lipoprotein cholesterol ratio (NHHR) is a new predictive indicator of atherosclerosis and dyslipidemia, which associated with increased risk of insulin resistance, diabetes, and prediabetes (10). The single point insulin sensitivity estimator (SPISE), calculated using TG, HDL-c, and body mass index (BMI), represents a reliable indicator for identifying insulin resistance in obese pediatric populations (13). However, investigations on the associations between plasma Mg and these novel glycolipid metabolism indicators are limited.

In the context of that, the cross-sectional research was conducted in Shenzhen, China aiming to preliminarily explore the association between plasma Mg levels and novel indicators of glucose metabolism (TyG, TyG-BMI, METS-IR, and SPISE), lipid metabolism (NHHR and LDL-c/HDL-c ratio), as well as to examine the association between Mg levels and metabolic diseases including diabetes and hyperlipidemia.

## 2 Materials and methods

### 2.1 Study population

**Figure 1:**
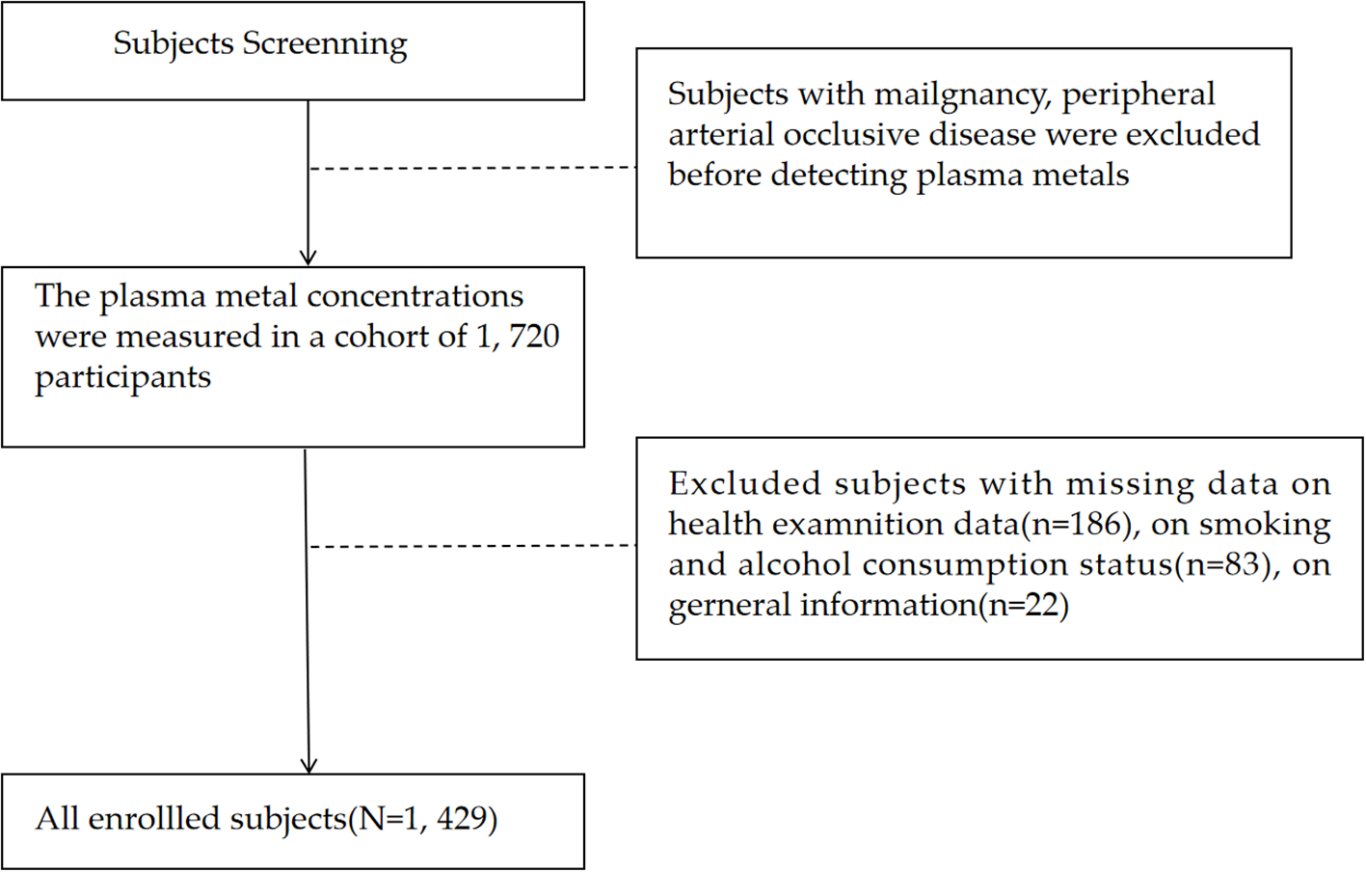
Flowchart of population selection.

All participants in our research were adults who received regular medical examinations at the Eighth Affiliated Hospital of Sun Yat-sen University in Shenzhen from October 2012 to April 2017. Participants with major diseases including neurological disorders, malignant tumors, cerebrovascular diseases, or peripheral arterial occlusive diseases were excluded. The plasma levels of Mg were detected among a total of 1,720 subjects above 30 years old using inductively coupled plasma mass spectrometry (ICP-MS) as mentioned previously (14). The participants with missing data on medical check-up data (n = 186), smoking status (n = 72), alcohol consumption status (n = 11) and the basic information (n = 22) were further excluded (**Figure 1**). Consequently, our study enrolled 1,429 adults. The research obtained approval from the Ethics Committee of the Shenzhen Center for Disease Control and Prevention. Every participant furnished informed consent prior to the start of the research. This cross-sectional study was conducted and reported in accordance with the Strengthening the Reporting of Observational Studies in Epidemiology (STROBE) guidelines.

### 2.2 General information and sample collection

With participants’ informed consents, trained healthcare professionals collected structured questionnaire data on age, sex, height, weight, lifestyle, and medical histories of diabetes and hypertension. Individuals who had smoked consistently for at least six months were classified as smokers, regardless of their current smoking status. Those having an alcohol intake of at least once a week for more than half a year were considered as alcohol drinkers. Trained technicians measured blood pressure, adhering to the World Health Organization’s definition of hypertension as a blood pressure ≥ 140/90 mmHg. After an overnight fast, each participant provided morning urine and blood samples. Venous blood samples were centrifuged at 4 °C, and the plasma was segregated and preserved at −80 °C until laboratory analysis. All biochemical indices analyzed in this study, including plasma Mg, fasting blood glucose, lipid profiles, and the biochemical parameters required for calculating glycolipid metabolism indices, were derived from the same morning fasting venous blood sample collected from each participant and were analyzed concurrently in the laboratory to ensure temporal consistency of the data.

### 2.3 Glucose indices and definition of diabetes

We employed four insulin resistance indicators included TyG, TyG-BMI, SPISE and METS-IR (15, 16). The formulas were as follows:


$$1.\text{TyG}=\text{Ln }\left[\text{TG}\times \text{FBG}\right]/2$$

  2. TyG‐BMI=TyG×BMI

$$3.\text{ METS}‐\text{IR}=\text{Ln }\left[2\times \text{FBG}+\text{TG}\right]\times \text{BMI}/\text{Ln }\left(\text{HDL}‐\text{c}\right)$$

$$4.\text{ SPISE}=600\times \text{HDL}‐{\text{c}}^{0.185}/\left(\text{T}{\text{G}}^{0.2}\times \text{BM}{\text{I}}^{1.338}\right)$$

BMI was calculated as weight (kg) divided by height squared (m²). FBG, TC, TG, and HDL-c in these formulas were measured in mg/dL. The criteria for diagnosing diabetes were as follows (17): FBG ≥ 7.0 mmol/L; using drugs for treating diabetes or self-admitted diagnosis of diabetes.

### 2.4 Lipid indices and definition of hyperlipidemia

Two novel blood lipid indicators NHHR and LDL-c/HDL-c ratio were included in this study (10). The formulas were as follows:


1. NHHR=non‐HDL‐c/HDL‐c

2. LDL‐c/HDL‐c ratio=LDL‐c/HDL‐c

The concentrations of LDL-c and HDL-c in these formulas were measured in mmol/L, converted from their original values in mg/dL. In line with the diagnostic criteria specified in the 2016 Chinese guideline for managing adult dyslipidemia, if the fasting blood lipid indicators of the subjects met any of the following criteria, it could be determined to be hyperlipidemia (18):


$$1.\text{ TC}\geq 5.2\;\left(200\right)\text{ mmol}/\text{L }\left(\text{mg}/\text{dL}\right)$$

$$2.\text{ TG}\geq 1.7\;\left(150\right)\text{ mmol}/\text{L }\left(\text{mg}/\text{dL}\right)$$

$$\;\;3.\text{ HDL}‐\text{c}<1.0\;\left(40\right)\text{ mmol}/\text{L }\left(\text{mg}/\text{dL}\right)$$

$$4.\text{ LDL}‐\text{c}\geq 3.4\;\left(130\right)\text{ mmol}/\text{L }\left(\text{mg}/\text{dL}\right)$$

### 2.5 Statistical analysis

All information of participants was presented as number with percentage for categorical variables, median with interquartile range or mean ± standard deviation for continuous variables. We employed the Chi-squared test for categorical variables. For continuous variables, we utilized either the Wilcoxon rank sum test or Student’s *t*-test to determine group differences. To investigate the association between plasma Mg and glycolipid metabolism indicators, the population was divided into quartiles based on plasma Mg concentration levels. Multivariable linear regression models were constructed to estimate the association between plasma Mg and the glycolipid indicators, illustrated by the coefficients of β and 95% confidence intervals (95% CIs). Model 1 incorporated age, sex, smoking status, and alcohol consumption status as adjusting factors. Age, sex, smoking status, alcohol consumption status, and ln-uric acid were included in Model 2. Building on Model 2, Model 3 added diabetes status and hypertension status. Variables that were not normally distributed underwent natural logarithm transformation to approximate a normal distribution, and those glycolipid indicators including TyG,TyG-BMI,SPISE,METS-IR,LDL-c,HDL-c,NHHR, and LDL-c/HDL-c were also transformed by natural logarithm. Furthermore, subgroup and sensitivity analyses were performed to assess the effects of potential confounders.

Restricted cubic spline (RCS) models were utilized in our analysis to examine the dose-response associations between plasma Mg, glycolipid metabolism markers, and metabolic disorders. The models were fit with 4 knots located at the 5th, 35th, 65th, and 95th percentiles of Mg, where the 50th percentile of Mg was used as the reference value. We used multiple logistic regression to analyze the relationships between plasma Mg and the risk of metabolic diseases, noted by the odds ratios (ORs) and 95% confidence intervals (CIs). Multivariable logistic regression models were constructed: Model 1 included age, sex, smoking status, and alcohol consumption status; Model 2 incorporated ln-BMI additionally to model 1; and model 3 further included hypertension status and ln-uric acid based on model 2.

Statistical analyses were performed with the use of R (“rcssci” package; version 4.4.2; Lucent Technologies, USA) and two-sided *p* < 0.05 were considered statistically significant.

## 3 Results

### 3.1 The characteristics of the study population

The average age was 59.6 years, and 43% of the participants were male. The median plasma Mg level was 0.86 mmol/L, and it was notably lower in the diabetic group (*p* < 0.05). Among the participants, 8.19%, 33.10% and 21.00% people was diagnosed with diabetes, hypertension, and hyperlipidemia, respectively. Novel glycolipid markers (TyG, TyG-BMI, METS-IR, NHHR and LDL-c/HDL-c) were significantly higher in the diabetic group (*p* < 0.001). The diabetic people also exhibited elevated levels of BMI, uric acid and FBG, while the level of HDL-c was lower (all *p* < 0.05) (**Table 1**).

**Table 1:** General characteristics of the study population.

Variables	Total population	Non-diabetics	Diabetics	*p-value*
	(N = 1,429)	(n = 1,252)	(n = 177)	
Magnesium, mmol/L	0.86 (0.79, 0.93)	0.86 (0.79, 0.93)	0.84 (0.77, 0.92)	< 0.050
Male, *n* (%)	610 (43.00)	532 (42.00)	78 (44.00)	
Age, years	59.60 ± 9.48	59.10 ± 9.49	63.40 ± 8.55	< 0.001
BMI, kg/m^2^	23.62 (21.79, 25.63)	23.55 (21.72, 25.55)	24.03 (22.21, 26.57)	0.004
UA, μmol/L	330.00 (280.00, 391.00)	328.00 (278.00, 389.00)	344.00 (299.00, 406.00)	0.013
Hypertension status, *n* (%)				< 0.001
No	956 (66.89)	880 (70.28)	76 (42.93)	
Yes	473 (33.10)	372 (29.71)	101 (57.06)	
Hyperlipidemia status, *n* (%)				0.001
No	1135(79.00)	1011 (81.00)	124 (70.00)	
Yes	294 (21.00)	241 (19.00)	53 (30.00)	
Smoking, *n* (%)	183 (12.80)	161 (12.85)	22 (12.42)	0.968
Alcohol drinking, *n* (%)	154 (10.77)	134 (10.70)	20 (11.29)	0.912
Glucose indicators				
FBG, mmol/L	5.45 (5.07, 5.97)	5.37 (5.03, 5.77)	7.62 (6.97, 9.19)	< 0.001
TyG	9.33 (8.94, 9.75)	9.27 (8.88, 9.64)	9.94 (9.48, 10.37)	< 0.001
TyG-BMI	221.1 (199.11, 244.03)	219.36 (197.65, 241.01)	240.17 (214.94, 268.31)	< 0.001
METS-IR	34.47 (30.65, 38.77)	34.09 (30.25, 38.33)	37.96 (33.23, 42.27)	< 0.001
SPISE	7.02 (5.97, 8.29)	7.1 (6.03, 8.39)	6.57 (5.45, 7.78)	< 0.001
Lipid indicators				
HDL-c, mmol/L	1.38 (1.16, 1.62)	1.40 (1.17, 1.63)	1.28 (1.08, 1.52)	< 0.001
LDL-c, mmol/L	3.08 (2.52, 3.72)	3.08 (2.52, 3.72)	3.11 (2.44, 3.70)	0.768
NHHR	2.88 (2.28, 3.61)	2.86 (2.25, 3.56)	3.16 (2.48, 3.81)	0.003
LDL-c/HDL-c	2.25 (1.78, 2.8)	2.24 (1.77, 2.79)	2.36 (1.84, 3)	0.028

Data were presented as number (percentage) for categorical data, mean ± standard deviation for parametrically distributed data or median (interquartile range) for non-parametrically distributed data. Student’s *t*-test or Wilcoxon rank sum test was used for comparison of the continuous variables according to the data distribution, and Chi-square test for the categorical variables.

*BMI*, body mass index; *UA*, uric acid; *FBG*, fasting blood glucose; *HbA1c*, glycated haemoglobin; *TyG*, the triglyceride-glucose index; *TyG-BMI*, the TyG index adjusted for BMI; *SPISE*, the Single Point Insulin Sensitivity Estimator; *METS-IR*, the Metabolic Score for Insulin Resistance total cholesterol; *HDL-c*, high-density lipoprotein cholesterol; *LDL-c*, low-density lipoprotein cholesterol; *NHHR*, the ratio of non-high-density lipoprotein cholesterol to high-density lipoprotein cholesterol.

### 3.2 The relationship between plasma levels of Mg and glycolipid metabolism indicators

To evaluate the influence of Mg levels on different glycolipid metabolism indicators, participants were divided into four groups by plasma Mg quartiles. For the glucose indicators in **Table 2**, the adjusted β (95% CI) for the highest quartiles of Mg were -0.010 (95%CI: -0.020, -0.001; p = 0.027) for TyG, -0.024 (95%CI: -0.045, -0.003; p = 0.025) for TyG-BMI in model 2, compared to the lowest quartile of Mg. However, after adjusting for diabetes status and hypertension status in model 3, the associations between the two indicators (TyG, TyG-BMI) and Mg were missing (All p >0.05). Negative associations between FBG and METS-IR and Mg levels were observed across all quartile groups. Compared to the lowest quartile of Mg, the adjusted β (95% CI) for the highest quartile of Mg was -0.043 (95%CI: -0.067, -0.020; p<0.001) for FBG in model 3 and -0.024 (95%CI: -0.046, -0.002; p = 0.034) for METS-IR in model 3.

**Table 2:** The association between quartiles of plasma Mg and glycolipid metabolism indicators.

Variables	Quartiles of plasma Mg						
	Q1 (n=358)	Q2 (n=357)		Q3 (n=357)		Q4 (n=357)	
		β (95%CI)	*p-value*	β (95%CI)	*p-value*	β (95%CI)	*p-value*
Indicators of glucose metabolism							
TyG							
Model 1	0 (ref)	-0.001 (-0.011, 0.009)	0.823	-0.005 (-0.014, 0.005)	0.341	-0.008 (-0.018, 0.001)	0.096
Model 2	0 (ref)	-0.005 (-0.015, 0.004)	0.245	-0.008 (-0.017, 0.002)	0.103	**-0.010 (-0.020, -0.001)**	0.027
Model 3	0 (ref)	-0.003 (-0.012, 0.006)	0.495	-0.005 (-0.014, 0.004)	0.265	-0.006 (-0.015, 0.003)	0.205
TyG-BMI							
Model 1	0 (ref)	-0.002 (-0.025, 0.020)	0.826	-0.007 (-0.029, 0.015)	0.546	-0.018 (-0.040, 0.005)	0.121
Model 2	0 (ref)	-0.015 (-0.035, 0.006)	0.161	-0.015 (-0.036, 0.005)	0.148	**-0.024 (-0.045, -0.003)**	0.025
Model 3	0 (ref)	-0.012 (-0.032, 0.008)	0.256	-0.010 (-0.031, 0.010)	0.309	-0.016 (-0.036, 0.004)	0.121
SPISE							
Model 1	0 (ref)	0.001 (-0.034, 0.035)	0.970	0.004 (-0.030, 0.039)	0.803	0.021 (-0.014, 0.055)	0.245
Model 2	0 (ref)	0.021 (-0.011, 0.053)	0.199	0.018 (-0.014, 0.050)	0.263	0.031 (-0.001, 0.063)	0.060
Model 3	0 (ref)	0.018 (-0.014, 0.049)	0.266	0.013 (-0.019, 0.044)	0.425	0.022 (-0.009, 0.054)	0.169
METS-IR							
Model 1	0 (ref)	-0.013 (-0.037, 0.012)	0.307	-0.015 (-0.039, 0.009)	0.218	**-0.027 (-0.051, -0.002)**	0.033
Model 2	0 (ref)	**-0.025 (-0.048, -0.003)**	0.030	**-0.024 (-0.047, -0.001)**	0.040	**-0.033 (-0.056, -0.010)**	0.005
Model 3	0 (ref)	-0.022 (-0.044, 0.000)	0.054	-0.019 (-0.041, 0.004)	0.101	**-0.024 (-0.046, -0.002)**	0.034
FBG							
Model 1	0 (ref)	**-0.049 (-0.079, -0.019)**	0.001	**-0.063 (-0.093, -0.033)**	0.001	**-0.072 (-0.102, -0.042)**	0.005
Model 2	0 (ref)	**-0.050 (-0.080, -0.020)**	<0.001	**-0.064 (-0.094, -0.034)**	<0.001	**-0.072 (-0.103, -0.042)**	<0.001
Model 3	0 (ref)	**-0.034 (-0.057, -0.010)**	<0.001	**-0.048 (-0.071, -0.024)**	<0.001	**-0.043 (-0.067, -0.020)**	<0.001
Indicators of lipid metabolism							
LDL-c							
Model 1	0(ref)	**0.072 (0.029, 0.116)**	0.001	**0.110 (0.066, 0.153)**	0.003	**0.046 (0.003, 0.090)**	0.003
Model 2	0(ref)	**0.066 (0.022, 0.109)**	<0.001	**0.105 (0.062, 0.149)**	<0.001	0.043 (-0.001, 0.087)	<0.001
Model 3	0(ref)	**0.066 (0.022, 0.109)**	0.038	**0.104 (0.060, 0.147)**	0.053	0.041 (-0.003, 0.085)	0.065
HDL-c							
Model 1	0(ref)	**0.035 (0.002, 0.067)**	0.036	0.029 (-0.004, 0.061)	0.082	**0.034 (0.001, 0.066)**	0.044
Model 2	0(ref)	**0.043 (0.011, 0.075)**	0.008	**0.035 (0.003, 0.067)**	0.034	**0.038 (0.006, 0.070)**	0.021
Model 3	0(ref)	**0.040 (0.008, 0.072)**	0.013	0.031 (-0.001, 0.063)	0.056	0.032 (-0.000, 0.064)	0.053
LDL-c/HDL-c							
Model 1	0(ref)	0.038 (-0.012, 0.088)	0.138	**0.081 (0.031, 0.131)**	0.002	0.013 (-0.038, 0.063)	0.618
Model 2	0(ref)	0.023 (-0.026, 0.072)	0.365	**0.071 (0.021, 0.120)**	0.005	0.005 (-0.044, 0.055)	0.838
Model 3	0(ref)	0.025 (-0.024, 0.075)	0.310	**0.073 (0.024, 0.122)**	0.004	0.009 (-0.040, 0.059)	0.709
NHHR							
Model 1	0(ref)	-0.046 (-0.100, 0.009)	0.100	-0.020 (-0.074, 0.035)	0.478	**-0.057 (-0.112, -0.002)**	0.041
Model 2	0(ref)	**-0.064 (-0.117, -0.011)**	0.018	-0.032 (-0.086, 0.021)	0.233	**-0.067 (-0.120, -0.013)**	0.015
Model 3	0(ref)	**-0.061 (-0.114, -0.008)**	0.024	-0.030 (-0.083, 0.024)	0.276	**-0.061 (-0.115, -0.008)**	0.025

Age, sex, smoking status, and alcohol consumption status were all included in Model 1; the factors in Model 1 and ln-uric acid were included in Model 2; the factors in Model 2 and diabetes status and hypertension status were included in Model 3. Results were standardized beta coefficients (β) with 95% Confidence Interval (CI) from multivariable linear regression, comparing Mg quartiles (Q1–Q4, Q1 as reference). For each quartile, the table showed the sample size (n). Statistical significance was set at *p-value* < 0.05 (two-tailed). Bold values indicated that the 95% confidence interval for the β coefficient did not include zero, which was equivalent to *p*<0.05 for the significance test of the coefficient. TyG, TyG-BMI, METS-IR, LDL-c, HDL-c, NHHR, and LDL-c/HDL-c were transformed by natural logarithm.

*TyG*, the triglyceride-glucose index; *TyG-BMI*, the TyG index adjusted for BMI; *SPISE*, the Single Point Insulin Sensitivity Estimator; *METS-IR*, the Metabolic Score for Insulin Resistance; *FBG*, fasting blood glucose; *LDL-c*, low-density lipoprotein cholesterol; *HDL-c*, high-density lipoprotein cholesterol; *NHHR*, the ratio of non-high-density lipoprotein cholesterol to high-density lipoprotein cholesterol.

For the lipid indicators, LDL-c and HDL-c showed positive associations with Mg levels across all quartile groups. Additionally, the adjusted β (95% CI) for the highest quartile of Mg was-0.057 (95%CI: -0.112, -0.002; p = 0.041) for NHHR in model 1, -0.067 (95%CI: -0.120, -0.013; p = 0.015) in model 2, and -0.061 (95%CI: -0.115, -0.008; p = 0.025) in model 3, compared with the lowest quartile of Mg. LDL-c/HDL-c index only showed positive relationship with Mg in the third quartile compared with the lowest quartile, the adjusted β (95% CI) of 0.081 (95%CI: 0.031, 0.131; p = 0.002) in model 1, 0.071 (95%CI: 0.021, 0.120; p = 0.005) in model 2, and 0.073 (95%CI: 0.024, 0.122; p = 0.004) in model 3.

### 3.3 Subgroup analysis and interaction effects

To account for potential confounding factors, a stratified analysis by age (> 60 years old, ≤ 60 years old) and gender (male, female) in the general population was conducted in the **Supplementary Figures S1**-**S4**. Age-stratified analysis showed a significant negative association between Mg and METS-IR in the ≤60-year-old group, but no significance in those over 60. The results of the interaction analysis showed that age could modify the association between Mg and METS-IR (*p*<0.05). Similarly, a positive association between Mg and LDL-c was found in the ≤60-year-old group, and the interaction analysis showed that age could also modify the association between Mg and LDL-c (*p*<0.05). The above findings demonstrated an interaction effect of age and Mg on glycolipid metabolic parameters. For gender stratification, no significant differences were observed between males and females in either group.

### 3.4 Sensitivity analysis

The sensitivity analysis was performed in non-diabetic participants (**Supplementary Table S1**). After adjustment, no significant associations were observed between Mg and the indices TyG, TyG-BMI, or METS-IR. The negative association between FBG and Mg remained unaffected by diabetes status, persisting as a significant inverse relationship. Positive correlations between Mg and LDL-c, as well as LDL-c/HDL-c, were identified; However, these associations became non-significant after adjustment for hypertension in Model 3. For HDL-c, compared to the first quartile, significant positive associations with Mg were observed only in the third and fourth quartile, with β (95% CI) of 0.036 (0.002, 0.070) in model 1, 0.035 (0.001, 0.069) in model 3 for the third quartile, and 0.036 (0.002, 0.070) in model 1 for the fourth quartile. For NHHR, compared to the first quartile, significant negative associations with Mg were observed only in the third and fourth quartiles, with β (95% CI) of -0.066 (-0.122, -0.010) in model 1, -0.064 (-0.120, -0.008) in model 3 for the third quartile, and -0.065 (-0.121, -0.009) in model 3 for the fourth quartile.

### 3.5 Dose-response relationship between plasma Mg levels and the glycolipid metabolism indicators

**Figure 2:**
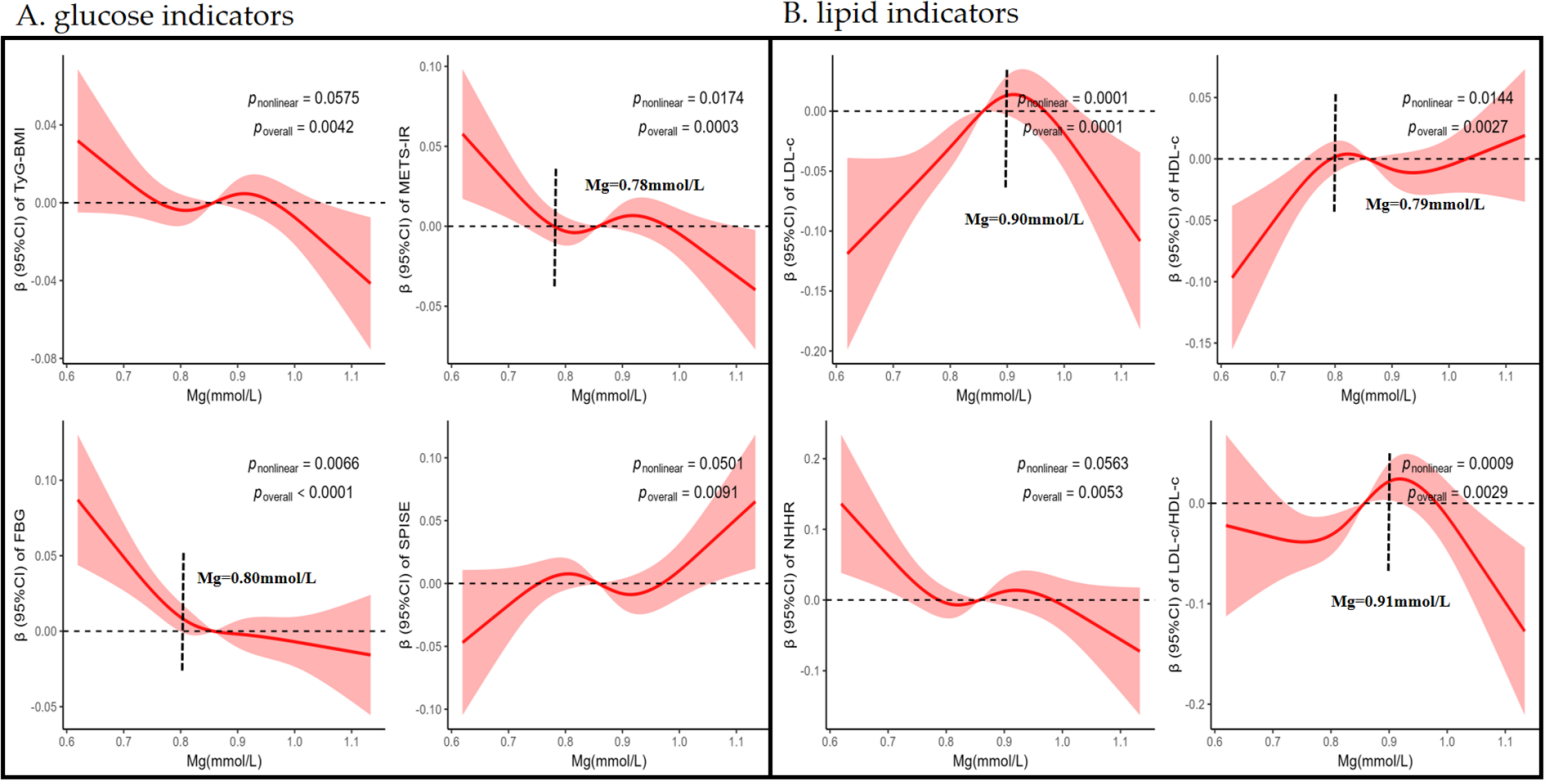
Restricted cubic spline (RCS) curve of the connection between plasma Mg and glycolipid metabolism indicators, including glucose indicators **(A)** and lipid indicators **(B)**. Results were presented as standardized beta coefficients (β) with 95% confidence intervals (CI), derived from restricted cubic spline models adjusted for age, sex, smoking status, alcohol consumption status, ln-uric acid, diabetes status and hypertension status. The inflection points on the curve indicated the plasma Mg concentration at which the direction or strength of the association changes. Corresponding *p-values* for the nonlinear and overall associations were displayed directly on the graph. TyG, TyG-BMI, METS-IR, LDL-c, HDL-c, NHHR, and LDL-c/HDL-c were transformed by natural logarithm. *TyG-BMI* the product of glucose and triglycerides(*TyG)* index adjusted for BMI, *METS-IR* the Metabolic Score for Insulin Resistance, *FBG* fasting blood glucose, *SPISE* the Single Point Insulin Sensitivity Estimator, *LDL-c* low-density lipoprotein cholesterol, *HDL-c* high-density lipoprotein cholesterol, *NHHR* the ratio of non-high-density lipoprotein cholesterol to high-density lipoprotein cholesterol, *LDL-c/HDL-c*, the ratio of low-density lipoprotein cholesterol to high-density lipoprotein cholesterol.

To investigate the uncovered association between Mg and glycolipid indicators, we used RCS model to explore it. For glucose indicators, negative and linear relationships were found between Mg and the indicators of TyG-BMI (p _nonlinear_ > 0.050 and p _overall_ < 0.050), but no significant association was found for TyG (p _overall_ > 0.050), as shown in **Supplementary Figure S5**. In contrast, Mg was positive linear correlated with SPISE (p _nonlinear_ > 0.050, p _overall_ < 0.050). Results revealed a negative non-linear relationship between Mg and FBG and METS-IR (p _nonlinear_ < 0.050, p _overall_ < 0.050), as shown in **Figure 2A**.

RCS analysis of the lipid indicators were shown in **Figure 2B**. A negative linear association was observed between Mg and NHHR (p _nonlinear_ > 0.050, p _overall_ < 0.050). LDL-c, HDL-c and LDL-c/HDL-c index exhibited a V-shaped nonlinear relationship with Mg (p _nonlinear_ < 0.050 and p _overall_ < 0.050).

Further threshold effect and piecewise regression analyses were performed on these nonlinear relationships (**Supplementary Figure S6**). The analysis revealed that Mg exhibited a threshold effect on METS-IR at 0.78 mmol/L (95% CI: 0.67–0.89). Below this threshold, each 1 mmol/L increase in Mg was marginally associated with a decrease in METS-IR (β= -0.307, p = 0.066), whereas above this level, the effect became non-significant. For FBG, a threshold point was identified at 0.80 mmol/L (95% CI: 0.72–0.88). Below this threshold, each 1 mmol/L increment in Mg corresponded to a significant reduction in FBG (β= -0.440, p = 0.003), while the effect attenuated to -0.046 (β= -0.046, p = 0.02) above this level. Regarding to the lipid metabolism indices, Mg demonstrated a threshold effect on LDL-c at 0.90 mmol/L (95% CI: 0.85–0.95). Below this cutoff, each 1 mmol/L increase in Mg was associated with an elevation in LDL-c (β= 0.540, p = 0.001), while above this threshold, the relationship reversed to a decrease (β= -0.530, p = 0.001). For HDL-c, the threshold was observed at 0.79 mmol/L (95% CI: 0.72–0.87), showing an increase in HDL-c per 1 mmol/L increment of Mg below this level (β= 0.540, p = 0.016), followed by a decreased effect (β= -0.015, p = 0.020) above the threshold. The LDL-c/HDL-c ratio exhibited a threshold at 0.91 mmol/L (95% CI: 0.85–0.97). Below this point, each 1 mmol/L Mg increase was linked to a rise in LDL-c/HDL-c (β= 0.330, p = 0.029), whereas above this threshold, a significant inverse association emerged (β= -0.590, p<0.001).

### 3.6 The relationship between plasma levels of Mg and diabetes

**Figure 3:**
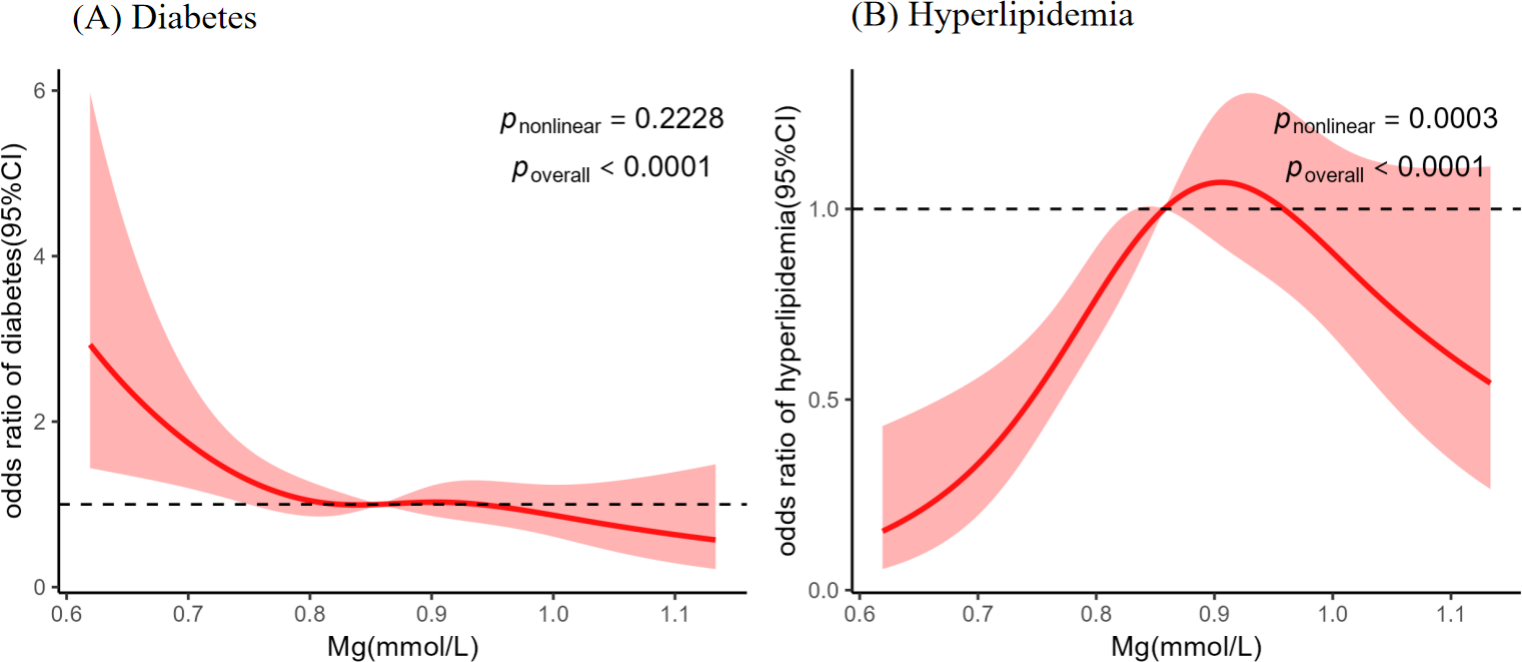
Restricted cubic spline (RCS) curve of the associations between plasma Mg and Diabetes **(A)**, Hyperlipidemia **(B)**. Results were presented as standardized beta coefficients (β) with 95% confidence intervals (CI). All models were adjusted for age, sex, smoking status, alcohol consumption status, ln-BMI, ln-uric acid, and hypertension status. The *p-values* for the nonlinear and overall associations were displayed on the graph.

As shown in **Figure 3A**, a negative linear association between Mg and diabetes was fitted using RCS model (*p*_nonlinear_ > 0.050, *p* _overall_ < 0.050). The results of the multivariate logistic regression models were consistent with the RCS model analysis, and in the highest quartile of Mg concentration, a significant reduction in the risk of diabetes (OR = 0.528, 95% CI: 0.329-0.848, *p* = 0.008) was observed, as shown in **Supplementary Table S2**.

### 3.7 The relationship between plasma levels of Mg and hyperlipidemia

As illustrated in **Figure 3B**, a significant non-linear association was observed between plasma Mg concentration and hyperlipidemia (*p*_nonlinear_ < 0.05, *p*_overall_ < 0.05). Below the threshold of 0.86 mmol/L, the relationship exhibited a positive trend. Further analysis using adjusted multivariate logistic regression models (**Supplementary Table S3**) revealed that individuals in the highest quartile of Mg concentration had a significantly elevated risk of hyperlipidemia compared to the lowest quartile (OR = 1.961, 95% CI: 1.279–3.008, *p* = 0.002).

## 4 Discussion

Numerous studies have verified the associations between novel glycolipid metabolism indicators and metabolic diseases, but the relationships between plasma Mg and these novel glycolipid metabolism indicators were largely unclear, and most of them only focused on dietary intake of Mg. This study represented the first systematic investigation of the associations between plasma Mg concentrations and glycolipid metabolic indices in a general population in Shenzhen. Mg demonstrated distinct linear associations with several glucose indicators, including the negative correlations with TyG-BMI, and a positive linear correlation with SPISE. Additionally, significant negative and non-linear relationships were observed between Mg and METS-IR, FBG. Additionally, Mg exhibited V-shaped non-linear correlations with the lipid parameters (LDL-c, HDL-c, and LDL-c/HDL-c ratio) and the negative relationship with NHHR. Mg was significantly negatively correlated with diabetes, while it showed a V-shaped non-linear correlation with hyperlipidemia. This study filled a research gap regarding the relationship between plasma Mg and novel glycolipid metabolism indicators, thereby providing new evidence for revealing the regulatory role of Mg in glycolipid metabolism.

Mg was a crucial element for the proper functioning of the human body, not only involved in the production of ATP and enzymes in the human body, but also in inflammatory responses (19). When the Mg concentration in the body was too low, it was more likely to cause inflammation, leading to the occurrence and development of metabolic diseases. In our study, 205 participants (14.34%) were identified as hypomagnesemia (<0.75mmol/L). Research indicated that hypomagnesemia was independently linked to increased risks of all-cause and cardiovascular mortality. Notably, while most people with borderline hypomagnesemia showed no obvious symptoms, long-term low Mg levels could still have a detrimental impact on physiological functions (20). In a Canadian cohort study, nearly 15% of adults were in a state of Mg deficiency (<0.75mmol/L), and low level of Mg was identified as a risk factor for diabetes and cardiovascular disease (21). Diabetic patients had a higher rate of hypomagnesemia compared to healthy individuals. Our study had 177 diabetic patients and Mg concentration in diabetic patients (median: 0.840mmol/L) was lower than that of the non-diabetic patients (median: 0.860mmol/L). A nested case-control study on diabetic patients in southern China revealed that Mg levels were significantly lower in individuals with diabetes (median: 0.860mmol/L) and pre-diabetes (median: 0.900mmol/L) compared to the control group (median: 0.980mmol/L) (22); In another cohort study, it was determined that individuals with diabetes had a greater risk of Mg deficiency than those without diabetes (23), and our study revealed consistent findings. This discrepancy was likely attributable to the pathological loss of Mg through urine (5). Mg deficiency was closely associated with the occurrence and development of metabolic diseases.

Numerous studies explored the links between traditional glycolipid metabolism indicators and metabolic diseases (24, 25), however, these traditional single indicators had limitations in evaluating various disease risk. New glycolipid metabolism indicators, which integrated multiple parameters such as TyG, TyG-BMI, SPISE, and METS-IR, might provide a more comprehensively reflection of glycolipid metabolic status by combining different metabolic risk factors into optimized formulas. TyG and TyG-BMI index, which included triglyceride, glucose levels and BMI, were a valid surrogate marker for insulin resistance as well as an autonomous predictor for identifying populations at early risk of cardiovascular disease, and both TyG and TyG-BMI showed a positive association with cardiovascular disease and its mortality (9). METS-IR was another novel insulin resistance index that was independent of fasting insulin levels and included some anthropometric indices (TG, FBG, BMI, and HDL-c), providing a simpler and more stable method for assessing insulin resistance. A prospective study involving over 6,000 participants verified that higher METS-IR levels correlated with an increased risk of diabetes (12). Numerous epidemiological studies validated the predictive utility of the SPISE index for assessing insulin resistance and related metabolic disorders. For instance, a cross-sectional investigation conducted within the Indian population demonstrated that SPISE index showed significantly higher diagnostic accuracy compared to conventional biomarkers in distinguishing insulin resistance from non-IR individuals (13). Higher SPISE index was independently associated with lower risk of future cardiovascular outcomes in type 2 diabetes patients after full adjustment for well-established metabolic confounders (26).

Notably, our study was the first to characterize the dose-response relationships between plasma Mg levels and the insulin resistance indices (TyG-BMI, METS-IR and SPISE), identifying both linear and nonlinear patterns. A significant negative linear relationship existed between Mg and TyG-BMI, while a positive linear association was observed with SPISE. Additionally, Mg showed an inverse non-linear relationship with METS-IR. Below 0.78 mmol/L, Mg exhibited a marginally significant inverse correlation with METS-IR (β = -0.307, *p* = 0.066), which became non-significant above this level, indicating reduced insulin resistance with rising Mg concentrations in hypomagnesemia, which means that supplementary of Mg for people with hypomagnesemia might alleviate the insulin resistance. These significant findings aligned with existing evidence suggested that increased Mg intake reduced insulin resistance and diabetes risk (27). Consistently, we also observed the significant inverse association between plasma Mg concentrations and diabetes incidence. Supporting these results, recent research demonstrated a negative correlation between Mg and insulin resistance (28). A segmented negative correlation between Mg and FBG came to light. Specifically, stronger inverse associations were observed when the Mg levels were lower than 0.80 mmol/L (β = -0.440, *p* = 0.003). However, these inverse association was weakened once the Mg levels exceeded this threshold (β = -0.046, *p* = 0.020), and this finding aligned with prior research (29). However, as causality remained unestablished, these associations require further validation through prospective cohort studies.

Regarding lipid metabolism, both linear and nonlinear patterns of interest emerged. This study identified a negative correlation between Mg and NHHR. Elevated NHHR may increase the risk of cardiometabolic multimorbidity (30). Mechanistically, this inverse association suggested that higher Mg concentrations might reduce cardiovascular disease risk by lowering non-HDL-c levels within the NHHR. Notably, we observed V-shaped trends between Mg levels and LDL-c, HDL-c, LDL-c/HDL-c ratio, with the threshold value set at 0.90 mmol/L, 0.79 mmol/L and 0.91 mmol/L of the level of Mg, respectively, for these indicators. Previous studies indicated that hypomagnesemia might induce dyslipidemia (elevated cholesterol and lipid levels) by suppressing HMG-CoA reductase activity, a key enzyme in cholesterol synthesis (31). Our analysis of threshold inflection points might raise doubts about the current diagnostic criterion for hypomagnesemia (< 0.75 mmol/L). Individuals with Mg levels within the conventional “normal range” may still exhibit clinical manifestations of Mg deficiency. These findings highlighted the clinical relevance of re-evaluating hypomagnesemia diagnostic criteria based on the results of lipid metabolism.

This cross-sectional study systematically explored the associations between plasma Mg levels and conventional and novel glycolipid metabolism indicators, diabetes, and hyperlipidemia in a relatively healthy cohort. The results confirmed the key role of Mg in regulating glycolipid metabolism, while also highlighting several limitations: the cross-sectional design restricted causal inference; only plasma Mg was measured, which may not fully reflect overall body Mg status (future studies should include broader measures like dietary intake); and the exclusive Shenzhen sample limited generalizability.

## 5 Conclusions

This study revealed linear and non-linear associations between plasma Mg and glycolipid metabolism indicators. With threshold analyses, it confirmed that hypomagnesemia predisposes individuals to glycolipid metabolic disorders. Future large-scale prospective studies are needed to validate our findings, which might provide important scientific evidence for the revision of current dietary guidance of Mg or the normal range of plasma Mg levels.

## Data Availability

The raw data supporting the conclusions of this article will be made available by the authors, without undue reservation.
