# Supplementary material for: Association between plasma magnesium levels and glycolipid metabolism in a southern Chinese population: a cross-sectional study in Shenzhen
Source: Front Endocrinol (Lausanne). 2026 Feb 24;17:1671459. doi: 10.3389/fendo.2026.1671459 (PMC12971470; doi:10.3389/fendo.2026.1671459)
Supplement: Supplementary file 1 [file DataSheet1.docx]

Supplementary Material

**Meilin Li^1†^, Die Hu^4†^, Ziyang Zou^3†^, Jiaxin Chen^1^, Dongju Zou^2^, Yanwei Zhang^3^, Jing-jun Han^5^, Jinling Liu^2^, Yanan Kuang^2^, Baosen Yan^2^, Jinquan Cheng^3^, Ziquan Lv^3^, Xiao Chen^1,3*^ and Suli Huang^2,3*^**

^1^School of Public Health, Shanxi Medical University, Taiyuan, China,

^2^School of Public Health, Shenzhen University Medical School, Shenzhen University, Shenzhen, China,

^3^Shenzhen Center for Disease Control and Prevention, Shenzhen, China,

^4^Immunization Section, Hefei Center for Disease Control and Prevention, Hefei, China,

^5^Department of Thoracic Surgery, The Eighth Affiliated Hospital, Sun Yet-Sen University, Shenzhen, China.

**^*^Correspondence:** grace420@szu.edu.cn (Suli Huang); [chenxiaoszcdc@aliyun.com](mailto:chenxiaoszcdc@aliyun.com) (Xiao Chen).

**^†^**Equal contribution and first authorship.

**Table of Contents**

**Table S1.** Associations of the quartile of plasma Mg concentration with glycolipid metabolism indicators in non-diabetic people.

**Table S2.** Associations of the plasma Mg concentration with diabetes status.

**Table S3.** Associations of the plasma Mg concentration with hyperlipidemia status.

**Figure S1.** Age-stratified analysis of Mg and glucose indicators.

**Figure S2.** Age-stratified analysis of Mg and lipid indicators.

**Figure S3.** Sex-stratified analysis of Mg and glucose indicators.

**Figure S4.** Sex-stratified analysis of Mg and lipid indicators.

**Figure S5.** Restricted cubic spline (RCS) curve of the connection between plasma Mg and TyG.

**Figure S6.** The threshold analysis of the non-linear relationship between Mg and glycolipid metabolism indices.

# Supplementary Tables

**Table S1.** Associations of the quartile of plasma Mg concentration with glycolipid metabolism indicators in non-diabetic people (N=1252).

| **Variables** | Quartiles of plasma Mg（mmol/L） | | | |
| --- | --- | --- | --- | --- |
|  | **Q1(0.438,0.786)** | **Q2(0.786,0.858)** | **Q3(0.858,0.932)** | **Q4(0.932,1.398)** |
|  | **(n=305)** | **(n=313)** | **(n=312)** | **(n=322)** |
| **Indicators of glucose metabolism** | | | | |
| TyG |  |  |  |  |
| Model 1 | 0 (ref) | 0.002 (-0.008, 0.011) | -0.002 (-0.012, 0.007) | -0.002 (-0.012, 0.007) |
| Model 2 | 0 (ref) | -0.001 (-0.011, 0.009) | -0.005 (-0.014, 0.005) | -0.005 (-0.014, 0.005) |
| Model 3 | 0 (ref) | -0.002 (-0.012, 0.008) | -0.004 (-0.014, 0.005) | -0.004 (-0.014, 0.005) |
| TyG-BMI |  |  |  |  |
| Model 1 | 0 (ref) | 0.008 (-0.015, 0.031) | -0.004 (-0.025, 0.018) | -0.004 (-0.025, 0.018) |
| Model 2 | 0 (ref) | 0.001 (-0.023, 0.024) | -0.010 (-0.031, 0.012) | -0.009 (-0.030, 0.013) |
| Model 3 | 0 (ref) | -0.008 (-0.031, 0.015) | -0.015 (-0.037, 0.006) | -0.013 (-0.035, 0.008) |
| SPISE |  |  |  |  |
| Model 1 | 0 (ref) | -0.011 (-0.048, 0.025) | 0.008 (-0.026, 0.041) | 0.008 (-0.026, 0.041) |
| Model 2 | 0 (ref) | -0.007 (-0.043, 0.030) | 0.010 (-0.024, 0.044) | 0.009 (-0.025, 0.042) |
| Model 3 | 0 (ref) | 0.011 (-0.026, 0.047) | 0.023 (-0.011, 0.056) | 0.020 (-0.013, 0.054) |
| METS-IR |  |  |  |  |
| Model 1 | 0 (ref) | -0.000 (-0.026, 0.025) | -0.013 (-0.036, 0.011) | -0.013 (-0.036, 0.011) |
| Model 2 | 0 (ref) | -0.007 (-0.033, 0.018) | -0.018 (-0.042, 0.006) | -0.017 (-0.041, 0.007) |
| Model 3 | 0 (ref) | -0.016 (-0.042, 0.009) | -0.024 (-0.048, -0.000) | -0.022 (-0.046, 0.001) |
| FBG |  |  |  |  |
| Model 1 | 0 (ref) | **-0.026 (-0.048, -0.004)** | **-0.027 (-0.049, -0.005)** | **-0.027 (-0.049, -0.005)** |
| Model 2 | 0 (ref) | **-0.054 (-0.076, -0.032)** | **-0.055 (-0.077, -0.033)** | **-0.054 (-0.076, -0.032)** |
| Model 3 | 0 (ref) | **-0.034 (-0.056, -0.012)** | **-0.035 (-0.057, -0.013)** | **-0.034 (-0.056, -0.012)** |
| **Indicators of lipid metabolism** | | | | |
| LDL-c |  |  |  |  |
| Model 1 | 0(ref) | **0.063 (0.017, 0.109)** | **0.057 (0.010, 0.103)** | **0.057 (0.010, 0.103)** |
| Model 2 | 0(ref) | **0.100 (0.054, 0.147)** | **0.095 (0.048, 0.141)** | **0.094 (0.048, 0.141)** |
| Model 3 | 0(ref) | 0.044 (-0.003, 0.090) | 0.040 (-0.007, 0.086) | 0.039 (-0.008, 0.085) |
| HDL-c |  |  |  |  |
| Model 1 | 0(ref) | 0.027 (-0.008, 0.062) | **0.036 (0.002, 0.070)** | **0.036 (0.002, 0.070)** |
| Model 2 | 0(ref) | 0.023 (-0.012, 0.058) | 0.031 (-0.003, 0.066) | 0.031 (-0.003, 0.065) |
| Model 3 | 0(ref) | 0.030 (-0.005, 0.064) | **0.035 (0.001, 0.069)** | 0.034 (0.000, 0.069) |
| LDL-c/HDL-c |  |  |  |  |
| Model 1 | 0(ref) | 0.036 (-0.018, 0.090) | 0.021 (-0.032, 0.074) | 0.021 (-0.032, 0.074) |
| Model 2 | 0(ref) | **0.077 (0.023, 0.131)** | **0.063 (0.010, 0.117)** | **0.063 (0.010, 0.117)** |
| Model 3 | 0(ref) | 0.014 (-0.040, 0.068) | 0.004 (-0.048, 0.057) | 0.004 (-0.049, 0.057) |
| NHHR |  |  |  |  |
| Model 1 | 0(ref) | -0.048 (-0.105, 0.009) | **-0.066 (-0.122, -0.010)** | **-0.066 (-0.122, -0.010)** |
| Model 2 | 0(ref) | -0.016 (-0.074, 0.041) | -0.032 (-0.088, 0.024) | -0.032 (-0.088, 0.024) |
| Model 3 | 0(ref) | -0.053 (-0.110, 0.004) | **-0.064 (-0.120, -0.008)** | **-0.065 (-0.121, -0.009)** |

Age, sex, smoking, and alcohol consumption were all included in Model 1; the factors in Model 1 and ln-uric acid were included in Model 2; the factors in Model 2 and hypertension were included in Model 3.

**Table S2.** Associations of the plasma Mg concentration with diabetes status.

| Varibles | N | Model1 |  |  | Model2 |  |  | Model3 |  |
| --- | --- | --- | --- | --- | --- | --- | --- | --- | --- |
|  |  | OR (95% CI) | *P-value* |  | OR (95% CI) | *P-value* |  | OR (95% CI) | *P-value* |
| Mg | 1429 | 0.095(0.019,0.449) | 0.003 |  | 0.099(0.020,0.476) | 0.004 |  | 0.099(0.021,0.473) | 0.004 |
| Quartile |  |  |  |  |  |  |  |  |  |
| Q1 | 358 | 1.00(Ref) |  |  | 1.00(Ref) |  |  | 1.00(Ref) |  |
| Q2 | 357 | 0.723(0.465, 1.122) | 0.149 |  | 0.700(0.448 ,1.094) | 0.118 |  | 0.697(0.446, 1.090) | 0.114 |
| Q3 | 357 | 0.713(0.460 ,1.107) | 0.133 |  | 0.734(0.471 ,1.145) | 0.173 |  | 0.733(0.470 ,1.142) | 0.171 |
| Q4 | 357 | 0.520(0.326 ,0.830) | 0.006 |  | 0.530(0.330, 0.850) | 0.008 |  | 0.528(0.329, 0.848) | 0.008 |

Age, sex, smoking status, alcohol consumption were included in Model 1; ln-BMI and the factors from Model 1 were included in Model 2; and the factors from Model 2 were combined with ln-uric acid and hypertension in Model 3. Q1, first quartile; Q2, second quartile; Q3, third quartile; Q4, fourth quartile.

**Table S3.** Associations of the plasma Mg concentration with hyperlipidemia status.

| Variables | N | Model 1 |  |  | Model 2 |  |  | Model 3 |  |
| --- | --- | --- | --- | --- | --- | --- | --- | --- | --- |
|  |  | OR (95% CI) | *P*-value |  | OR (95% CI) | *P*-value |  | OR (95% CI) | *P*-value |
| Mg | 1429 | 5.869（2.000，17.218） | 0.001 |  | 6.002（2.004，17.627） | 0.001 |  | 5.620（1.887，16.732） | 0.002 |
| Quartile |  |  |  |  |  |  |  |  |  |
| Q1 | 358 | 1.00(Ref) |  |  | 1.00(Ref) |  |  | 1.00(Ref) |  |
| Q2 | 357 | 2.392（1.577，3.628） | <0.001 |  | 2.383（1.570，3.617） | <0.001 |  | 2.257（1.482，3.438） | <0.001 |
| Q3 | 357 | 2.678（1.772，4.046） | <0.001 |  | 2.704（1.789，4.088） | <0.001 |  | 2.620（1.728，3.974） | <0.001 |
| Q4 | 357 | 2.011（1.316，3.072） | <0.001 |  | 2.030（1.329，3.103） | 0.001 |  | 1.961（1.279，3.008） | 0.002 |

Age, sex, smoking status, alcohol consumption were included in Model 1; ln-BMI and the factors from Model 1 were included in Model 2; and the factors from Model 2 were combined with ln-uric acid and hypertension in Model 3. Q1, first quartile; Q2, second quartile; Q3, third quartile; Q4, fourth quartile.

# Supplementary Figures

**
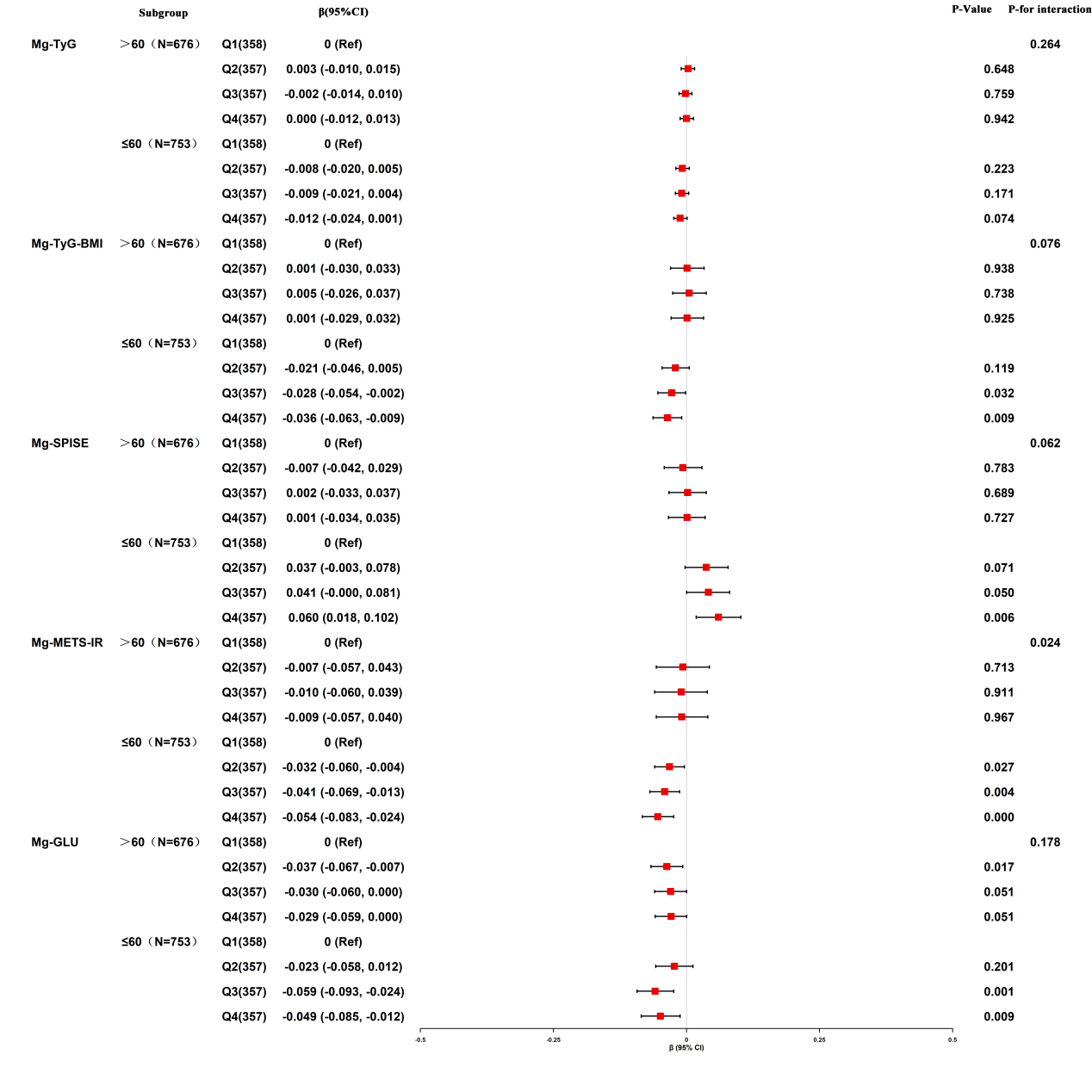
**

**Figure S1.** Age-stratified analysis of Mg and glucose indicators. Adjusted for the covaribles including sex, ln-uric acid, hypertension, diabetes, smoking and drinking.

**
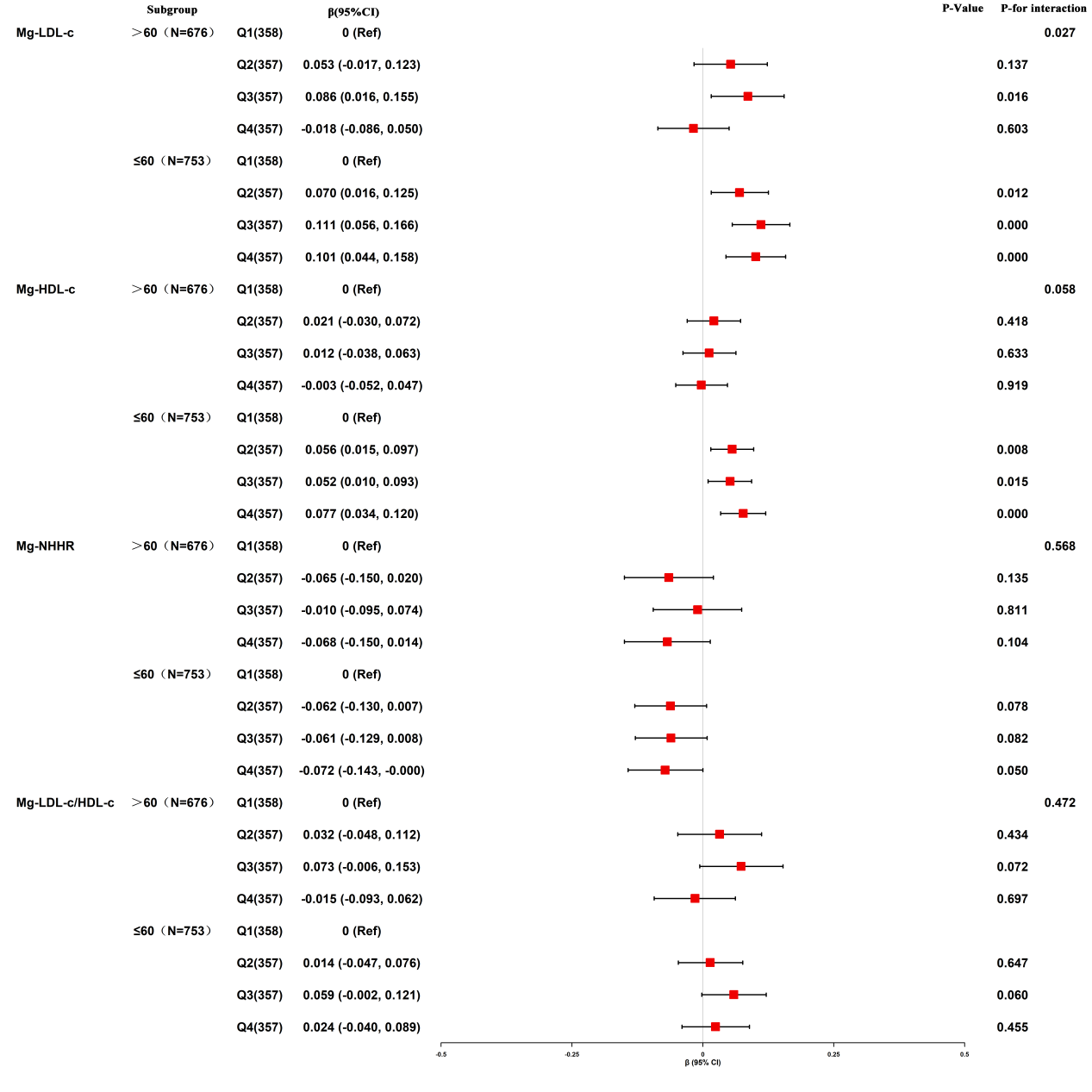
**

**Figure S2.** Age-stratified analysis of Mg and lipid indicators. Adjusted for the covariables including sex, ln-uric acid, hypertension, diabetes, smoking and drinking.

**
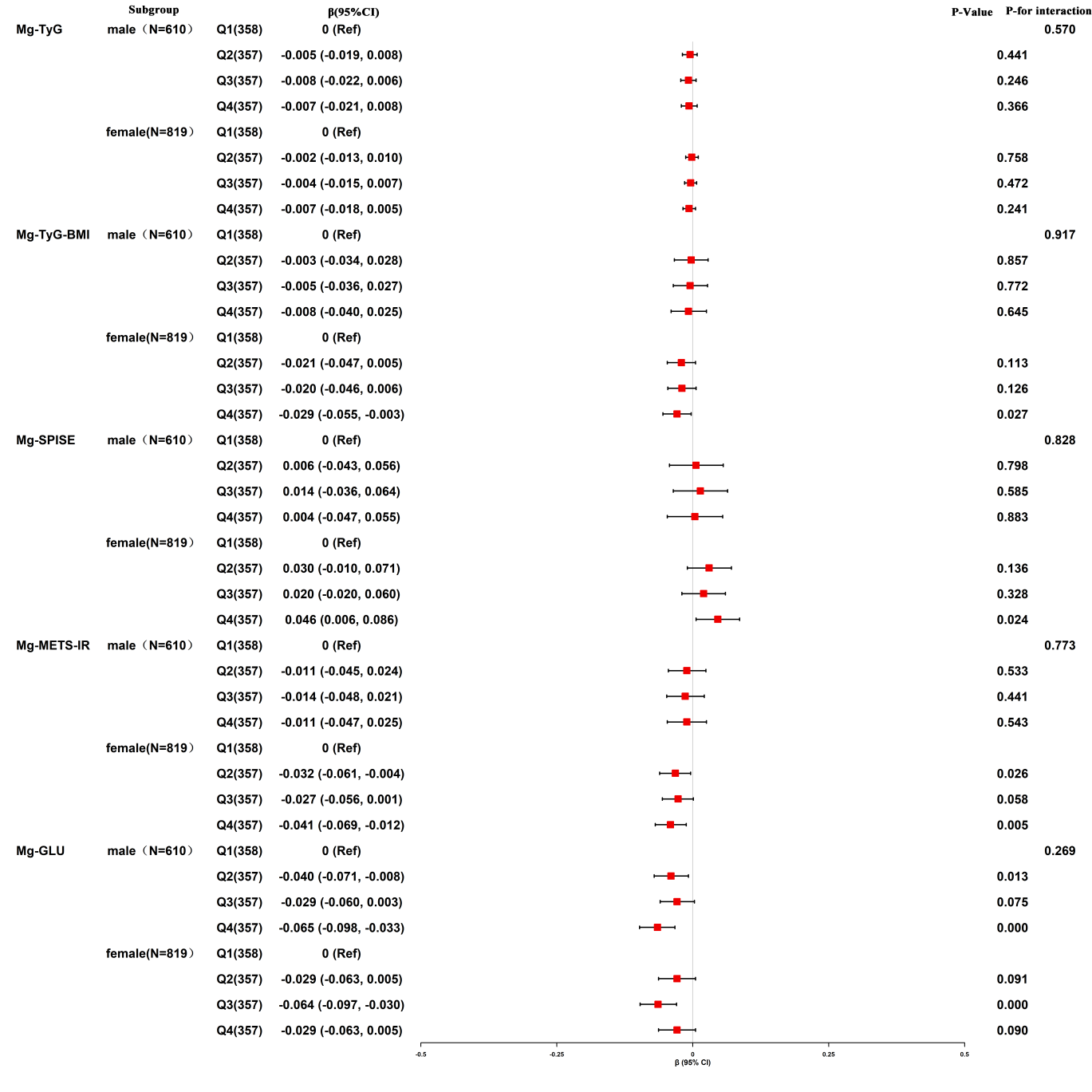
**

**Figure S3.** Sex-stratified analysis of Mg and glucose indicators. Adjusted for the covariables including age, ln-uric acid, hypertension, diabetes, smoking and drinking.

**
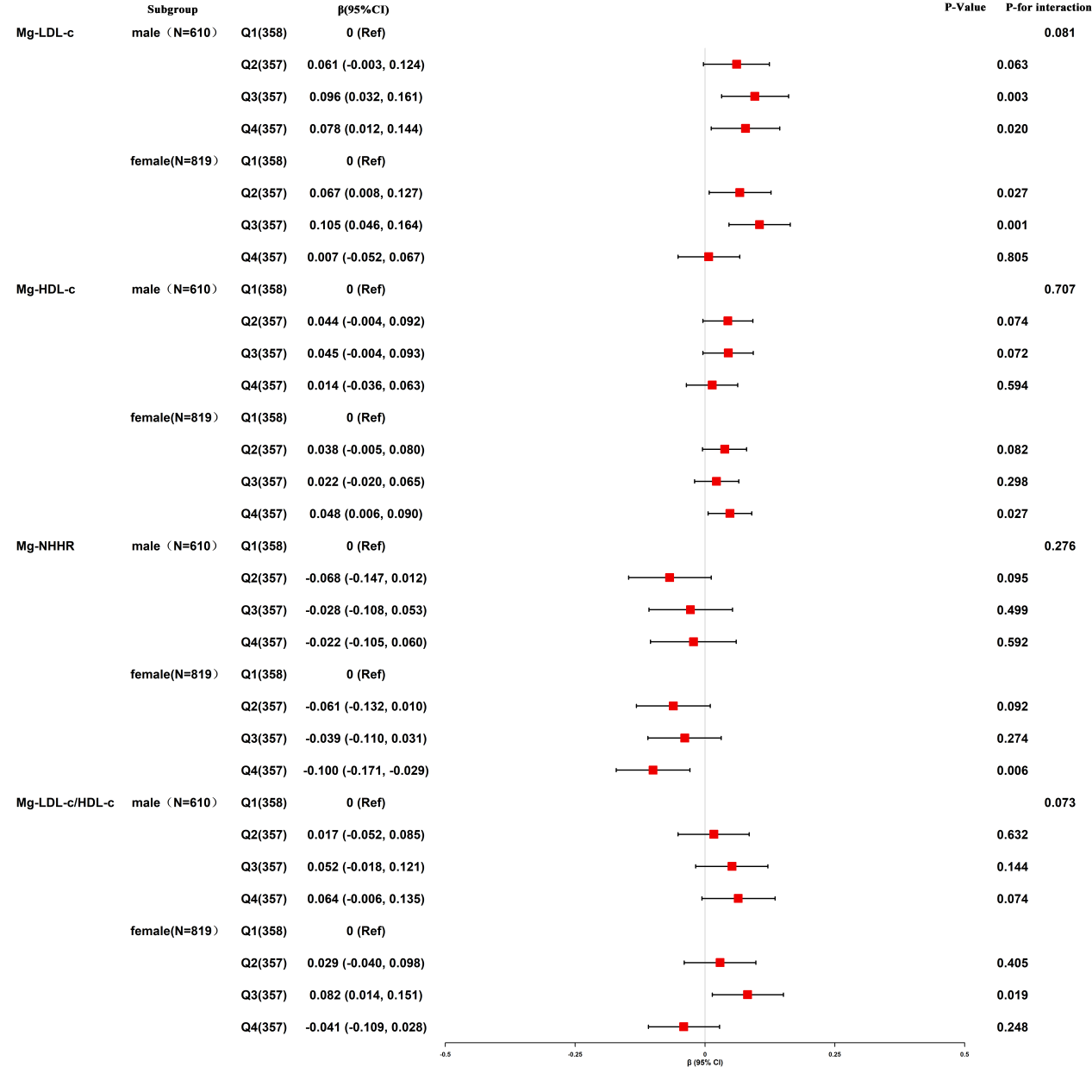
**

**Figure S4.** Sex-stratified analysis of Mg and lipid indicators. Adjusted for the covariables including age, ln-uric acid, hypertension, diabetes, smoking and drinking.

**
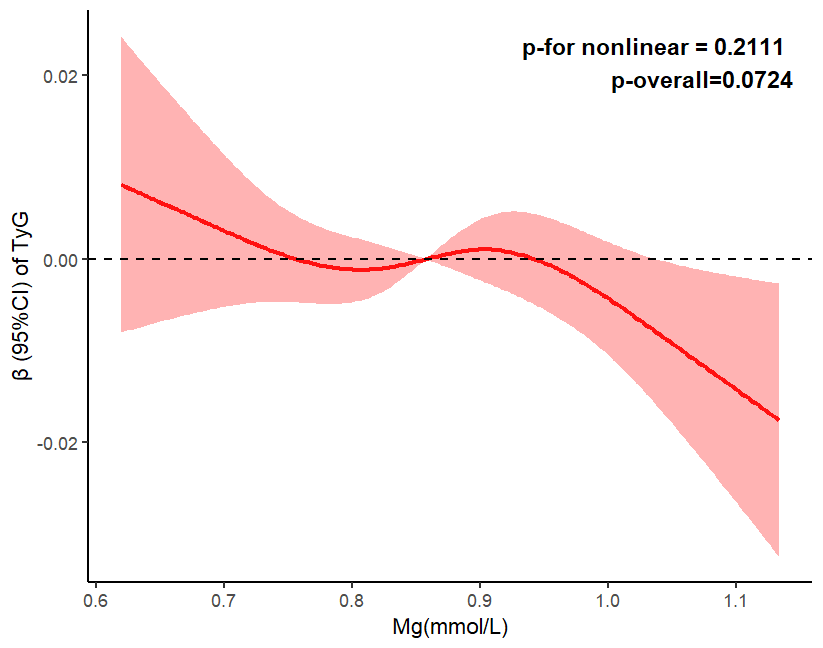
**

**Figure S5.** Restricted cubic spline (RCS) curve of the connection between plasma Mg and TyG. Adjusted for age, sex, smoking status, alcohol consumption, ln-uric acid, hypertension status and diabetes status.


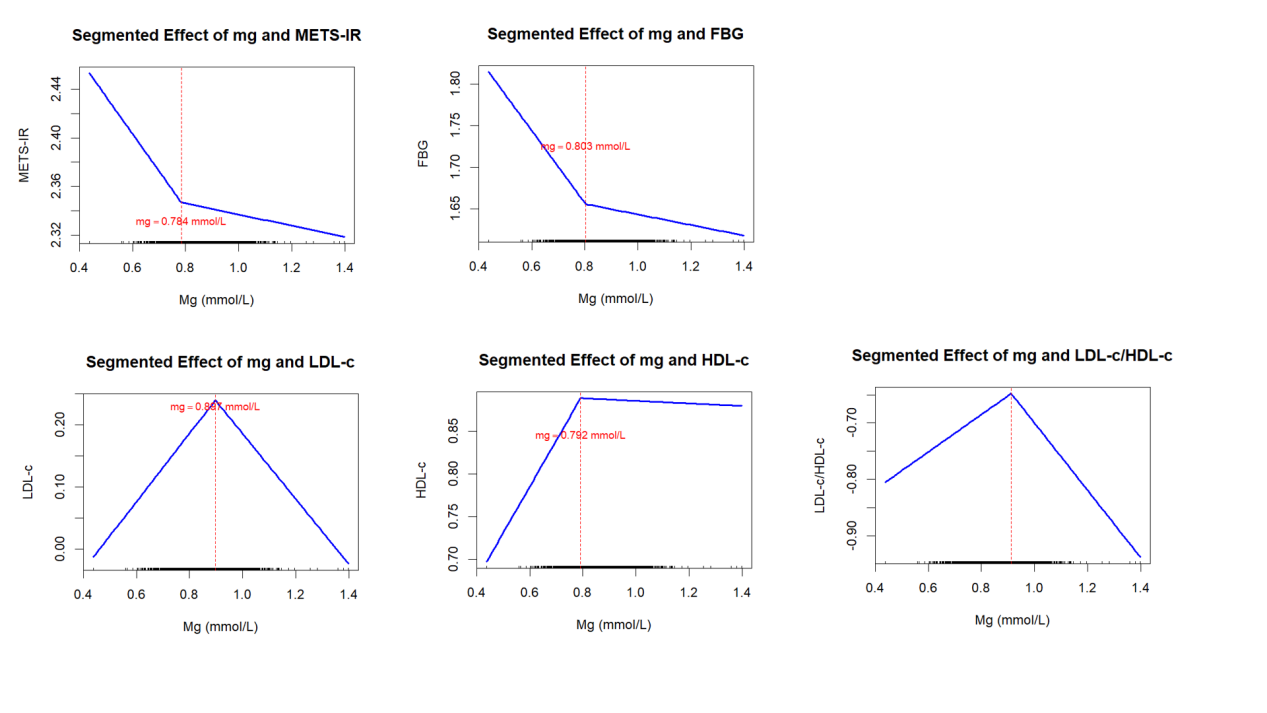


**Figure S6.** The threshold analysis of the non-linear relationship between Mg and glycolipid metabolism indices. The models were adjusted for age, sex, smoking, drinking, ln-uric acid, hypertension, and diabetes status.
